# Metagenomic Exploration Uncovers Several Novel ‘*Candidatus*’ Species Involved in Acetate Metabolism in High‐Ammonia Thermophilic Biogas Processes

**DOI:** 10.1111/1751-7915.70133

**Published:** 2025-03-24

**Authors:** George B. Cheng, Erik Bongcam‐Rudloff, Anna Schnürer

**Affiliations:** ^1^ Department of Molecular Sciences Swedish University of Agricultural Sciences Uppsala Sweden; ^2^ Department of Animal Biosciences Swedish University of Agricultural Sciences Uppsala Sweden

**Keywords:** acetogen, high‐ammonia, metagenomics, syntrophic acetate‐oxidising bacteria, *Thermodarwinisyntropha*, thermophilic, *Thermosyntrophaceticus*, *Thermotepidanaerobacter*

## Abstract

Biogas reactors operating at elevated ammonia levels are commonly susceptible to process disturbances, further augmented at thermophilic temperatures. The major cause is assumed to be linked to inhibition followed by an imbalance between different functional microbial groups, centred around the last two steps of the anaerobic digestion, involving acetogens, syntrophic acetate oxidisers (SAOB) and methanogens. Acetogens are key contributors to reactor efficiency, acting as the crucial link between the hydrolysis and fermentation steps and the final methanogenesis step. Their major product is acetate, at high ammonia levels further converted by SAOB and hydrogenotrophic methanogens to biogas. Even though these functionally different processes are well recognised, less is known about the responsible organism at elevated temperature and ammonia conditions. The main aim of this study was to garner insights into the penultimate stages in three thermophilic reactors (52°C) operated under high ammonia levels (FAN 0.7–1.0 g/L; TAN 3.6–4.4 g/L). The primary objective was to identify potential acetogens and SAOBs. Metagenomic data from the three reactors were analysed for the reductive acetyl‐CoA pathway (Wood–Ljungdahl Pathway) and glycine synthase reductase pathway. The results revealed a lack of true acetogens but uncovered three potential SAOB candidates that harbour the WLP, ‘*Candidatus* Thermodarwinisyntropha acetovorans’, ‘*Candidatus* Thermosyntrophaceticus schinkii’, ‘*Candidatus* Thermotepidanaerobacter aceticum’, and a potential lipid‐degrader ‘*Candidatus* Thermosyntrophomonas ammoiaca’.

## Graphical abstract

Metagenomic analysis of three thermophilic species was performed to identify key species for stable biogas production at high free ammonia levels (> 0.7 g/L). Potential acetogens and syntrophic acetate‐oxidising bacteria (SAOB) were recovered by looking for genes of the Wood–Ljungdahl pathway and glycine synthase reductase pathway.

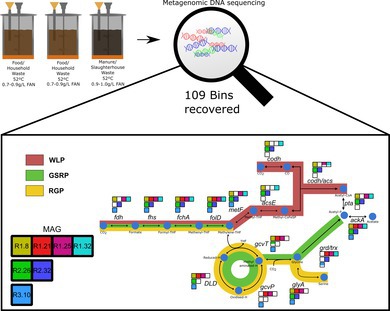

## 1 Introduction

Anaerobic digestion (AD) is an important process for the bio‐based economy as it can be used as a method for valorisation of organic waste and production of energy (biogas) and a digestate, suitable to use as an organic fertiliser in agriculture (Kougias and Angelidaki 2018). AD provides an effective strategy to combat the environmental issues of today: climate change, eutrophication, acidification, and air pollution (Paolini et al. 2018; Aghel et al. 2022). To obtain a well‐functioning process with high degradation efficiency of organic material and biogas production, as well as a high‐quality fertiliser, many different parameters need to be considered, both the composition of ingoing material as well as different operational parameters such as organic load (OLR), hydraulic retention time (HRT) and operational temperature (Ahlberg‐Eliasson et al. 2017; Sarker et al. 2019).

Among different materials used in AD processes, the ones with high protein content, such as food wastes and slaughterhouse waste, are especially noteworthy due to their abundance and potential for high biogas yield and contribute high nutrient value for the fertilisers (Chiew et al. 2015; Koszel and Lorencowicz 2015; Ehmann et al. 2018). However, such materials also present operational challenges if not carefully managed. Degradation of proteins results in the release of ammonium; at low concentrations, providing nitrogen essential for cell growth; however, at high concentrations, it is inhibitory for microbial activities (Gallert et al. 1998). The inhibitory effects are accentuated with increasing temperature and pH, as this shifts the equilibrium towards ammonia, which is the main toxic compound (Angelidaki and Ahring 1993; Gallert et al. 1998). For this reason, thermophilic biogas processes (> 50°C) are more prone to disturbances as compared to their mesophilic counterparts (Appels et al. 2008; Niu et al. 2015; Gebreeyessus and Jenicek 2016). Additionally, hydrogen sulphide produced during the degradation of proteins can also cause inhibitions, direct or indirect, by the precipitation of trace metals that are essential for microbial activity (Barton et al. 2014). Combined with ammonia inhibition, this can be the cause of severe process disturbances. To mitigate risk for inhibition, many biogas plants operating with proteinaceous materials add iron, alone or combined with trace metals. This can support microbial activities and process stability (Baek et al. 2019; Vu et al. 2022). Ammonia inhibition problems associated with temperature are rather unfortunate as the thermophilic compared to mesophilic operational temperatures have many advantages, such as potential for higher degradation and methane production rates, in situ sanitation, higher solubility of organic materials, and lower viscosity, giving less energy cost for stirring etc. (Labatut et al. 2014). Thus, the positive benefits of operating at thermophilic temperatures must be weighed against the risks of the process becoming more susceptible to disturbances.

The AD process consists of four interlinked microbial degradation steps: hydrolysis, acidogenesis, acetogenesis, and methanogenesis (Adekunle and Okolie 2015; Meegoda et al. 2018). These steps are represented by four functional groups of microorganisms, often with intra‐dependent metabolic pathways that are crucial to the performance of the AD process (Adekunle and Okolie 2015). Errant changes occurring in any group can affect the efficiency of the whole digestion process. Hydrolysis is the initial step that breaks down complex organic macromolecules into monomeric compounds, which in the following step are fermented into different organic acids, alcohols, carbon dioxide, and hydrogen. The third step involves further conversion of different organic acids via acetogenesis into acetate and hydrogen/carbon dioxide, key intermediates for the final step: methanogenesis. For thermodynamic reasons, the conversion of different organic acids can only proceed at low partial pressure of hydrogen, maintained via a syntrophic relationship with the methanogenic community (Schink 1997; Westerholm et al. 2022). Methane can be generated through two main pathways: direct conversion of hydrogen/carbon dioxide (or formate) to methane via hydrogenotrophic methanogenesis (HM), or cleavage of acetate to methane and carbon dioxide by acetoclastic methanogenesis (AM). An alternative route proceeds via syntrophic acetate oxidation (SAO), which involves a relationship between a syntrophic acid‐oxidising bacterium (SAOB), converting acetate into hydrogen and carbon dioxide/formate, to be used by methanogens via HM for the production of methane (Westerholm et al. 2011, 2016). SAO typically becomes the dominant pathway for methanogenesis from acetate at high ammonia levels and/or elevated temperatures, overtaking AM, which is more sensitive to ammonia than HM (Hao et al. 2021). Under these conditions, the activity of HM is critical to maintain low hydrogen partial pressure and thus facilitate syntrophic acid oxidation. This function is essential to prevent disruptions in the preceding step, which could otherwise cause an accumulation of VFAs and potentially lead to process failure (Westerholm et al. 2016).

As mentioned above, acetogens play a key function in the process. This group contains facultative autotrophs that can grow both as heterotrophs oxidising a wide range of organic substrates, or as chemolithotrophs using inorganic substrates such as hydrogen, carbon monoxide, and carbon dioxide, which are referred to as homoacetogens (Drake 1994). This flexibility in metabolism to convert a variety of substrates to acetate provides the link between the fermentative bacteria and methanogens (Diekert and Wohlfarth 1994; Karekar et al. 2022). In various anoxic environments, the acetogens compete with primary and secondary fermenters (Schuchmann and Müller 2016). Since conversion of glucose via acetogenesis generates a higher energy yield as compared to glucose‐dependent fermentation, acetogens should in theory have a competitive advantage (Drake et al. 2006). The pathway is used as (1) a mechanism for reductive synthesis of acetyl‐CoA from CO_2_, (2) a terminal electron‐accepting, energy‐conserving process, and (3) a process for CO_2_ fixation in cellular carbon synthesis (Drake 1994). Based on this definition, the ability to produce acetate is not critical to the identity of acetogens, but rather the process in which acetyl‐CoA is synthesised. The WLP is considered the most energetically efficient pathway for fixing CO_2_ into formate and acetyl‐CoA. Interestingly, the WLP is a reversible pathway and can also be utilised in the oxidative direction, as proposed for some SAO bacteria (SAOB), such as the thermophilic SAOB, *Thermoacetogenium phaeum*, able to run the pathway in both directions (Oehler et al. 2012). Moreover, a growing body of evidence proposes an alternative pathway to be in functional cooperation with the WLP for autotrophic growth of acetogens and during acetate oxidation by SAOBs (Song et al. 2020), i.e. the Glycine Cleavage System (GCS), which is a part of the Glycine Synthase Reductase Pathways (GSRP) and the Reductive Glycine Pathway (RGP). In the GSRP, CO_2_ is instead converted to acetate via glycine and acetyl‐phosphate (Fuchs 1986; Bar‐Even et al. 2012).

The interactionsbetween the functional microbes involved in acetogenesis, SAO, and HM represent a key for maintaining an efficient and stable process (Yin et al. 2018; Lim et al. 2020). Operation of AD at elevated temperature/ammonia levels represents a challenge in this regard as it typically risks an imbalance between these steps. Still, operation under this condition is possible with careful management of the process. Even so, the knowledge about thermophilic ammonia‐tolerant acetogens and SAOBs remains scarce. As of this publication, few acetogens have in general been recovered from the biogas process, and moreover, only a few characterised acetogens are thermophilic. However, there are many documented acetogens in many other natural environments, such as soil, water sediment, and gut systems (Drake et al. 2008; Kim et al. 2023). This study aims to shed some light on the bacterial groups that contribute to stable biogas production under high‐ammonia/temperature conditions and, in addition, to identify potentially new genomes of acetogens and SAOBs from biogas processes. Metagenomic data was retrieved from three different thermophilic high ammonia (> 3.6 g/L) lab‐scale continuously stirred tank reactors operating with municipal food waste or a mix of slaughterhouse waste and swine manure. To identify the potential acetogens/SAOBs, metagenomic‐assembled genomes (MAGs) were screened for genes coding for the WLP/GSRP enzymes, including key functional markers, i.e., formyltetrahydrofolate synthetase (*fhs*) and carbon‐monoxide dehydrogenase/acetyl‐CoA synthase (*codh*/*acs*).

## 2 Experimental Procedures

### 2.1 Reactors

Three laboratory‐scale continuously stirred tank reactors (CSTR; Belach Bioteknik, Stockholm, Sweden), with a working volume of 5 L and stirring speed of 90 rpm, were operated under thermophilic temperature (52°C) and with semi‐continuous feeding (6 days a week; Table 1). R1 and R2 were included from a previously published study in which they were designated as *D*_inc_ and *D*_52_ (Westerholm et al. 2018). Both these reactors were operated with source‐sorted organic household waste and waste from the food industry, including slaughterhouse waste, representing 80%–85% and 15%–20% on a VS basis, respectively. The substrate mixes were similar but not identical as they were collected from two different municipalities with biogas plants (Westerholm et al. 2018). Reactor R3 was operated with a mix of swine manure and slaughterhouse waste (50/50 on VS basis). The organic loading rate and HRT were similar for all reactors, at 2.0–3.0 g VS/L/day and 29–40 days, respectively (Table 1). The total time of operation under these conditions was 170, 80, and 120 days, for R1, R2, and R3, respectively. All reactors were supplemented with iron and, in the case of R1 and R2, with additional trace metals (Westerholm et al. 2016). R1 and R2 received 2.5 and 1.5 mL/kg substrate of Kemira BDP‐865/866 (Kemira Oyj, Helsingborg, Sweden) and R3 was supplemented with 6.25 g of iron‐rich sludge/kg of substrate (Persson et al. 2017). All lab reactors simulated the full‐scale processes and thus substrates and iron supplements were collected at the industrial site. The total free ammonia (FAN) level in all reactors ranged from 0.7 to 1.0 g/L. Total ammonia nitrogen (TAN) was between 3.7 and 4.4 g/L in R1 and R2, while in R3 it was 3.6 g/L. All reactors had low levels of VFA (< 0.5–1.5 g/L), with the highest value for R3.

**TABLE 1:** Operation and performance parameters for reactors R1, R2 and R3.

Reactor	R1	R2	R3
Temperature (°C)	52	52	52
pH	8.10 ± 0.02	8.10 ± 0.05	7.98 ± 0.12
Substrate	Household waste Food industry waste	Household waste Food industry waste	Swine manure Slaughterhouse waste
Working volume (L)	5	5	5
Organic loading rate (g VS/L/day)	2.5	3.0	2.0
Hydraulic retention time (days)	40	35	29
Specific methane production (SMP) NmL (CH_4_/g VS)	522 ± 15	615 ± 12	543 ± 15
Free ammonia nitrogen (g/L)	0.7–0.9	0.7–0.9	0.8–1.0
Total ammonia nitrogen (TAN) (g/L)	3.7–4.4	3.7–4.4	3.6
VFA (g/L)	< 0.5	< 0.5	1–1.5

### 2.2 Extraction and Metagenomic Analysis

Samples were collected from the three reactors at the end of the operational phase, and DNA was extracted with the FastDNA soil kit (MP Biomedicals, France) following the provided protocol, with an additional wash step with humic acid before the addition of the SEWS‐M step (Danielsson et al. 2017). DNA was sent to Eurofins GATC Biotech GmbH for library preparation and Illumina MiSeq sequencing (Project NG‐17000). Reads generated were filtered with TrimGalore (v. 0.6.1) removing adapters and low‐quality reads (Phred score ≤ 33). Assembly was completed using MegaHit (v. 1.2.9). Bins were generated and sorted into MAGs using MetaBat2 (v. 2.12.1). CheckM (v. 1.1.3) was used to assess the quality of MAGs, classifying high quality (≥ 90% completeness, ≤ 10% contamination), medium quality (≥ 70% completeness, > 10% contamination), and low quality (< 70% completeness, > 10% contamination). Classification of the raw reads was completed with Kraken2 (v. 2.1.2‐20211210‐4f648f5) and visualised with Krona (v. 2.7.1). GTDB‐Tk (v. 1.5.0) was used to retrieve taxonomic classification of the MAGs. EZBioCloud ANI Calculator (Yoon et al. 2017) and GGDC v3.0 (Meier‐Kolthoff et al. 2022) were used to estimate the average nucleotide identity (ANI) and digital DNA–DNA hybridisation (dDDH) between MAGs and classified genomes from GTDB‐Tk. Automatic annotation of genes encoding proteins on each MAG was performed using Prokka (v. 1.12‐12547ca) and functional gene identification was investigated with EggNOG (v. 2.1.9). AcetoBase (v 2.2) was used in conjunction with the gene prediction tools to determine which bin possessed *fhs* for selection for further analysis. Estimation of relative abundance of each MAG was performed by mapping the raw reads against the MAG using BWA (v. 0.7.17) with default parameters. The “pileup.sh” script from BBmap (v. 38.26) was used to generate the information about the mapped reads. The relative abundance of each MAG was calculated using the total number of reads that mapped to the MAGs and then divided by the total number of reads recovered in the corresponding reactor sample.

### 2.3 MAG Selection and Screening for Wood–Ljungdahl and Glycine Synthase Reductase Pathway and Pathway Analysis

Bins were categorised into three groups based on quality: high, medium, and low. The high‐quality bins were considered as MAGs for this study. All bins were screened for the *fhs* gene using Prokka and eggNOG and supplemented with corroboration from Acetobase (v2; Singh and Schnürer 2021). The bins possessing *fhs* were further investigated for the rest of the Wood–Ljungdahl pathway genes encoding methylenetetrahydrofolate dehydrogenase/methenyltetrahydrofolate cyclohydrolase (*folD*), formate dehydrogenase (*fdhA*), methylenetetrahydrofolate reductase (*metF*), acetyl‐CoA decarbonylase/synthase, CODH/ACS complex subunit gamma and delta (*acsCD*), acetyl‐CoA synthase (*acsB*), anaerobic carbon‐monoxide dehydrogenase catalytic subunit (*acsA*/*cooS*), and 5‐methyltetrahydrofolate corrinoid/iron–sulphur protein methyltransferase (*acsE*). The enzyme was considered fully recovered if all the genes encoding all the subunits of the enzyme were present. If one or more subunits were missing, the gene was regarded as partially recovered. Upon the discovery of the absence of *codh/acs*, the medium‐ and low‐quality bins and unbinned reads were also screened to determine if the gene was missing or not binned. All bins were then screened for genes encoding the glycine synthase reductase pathway: *grd*, *gcvPA*, *gcvT*, *lpdA*. The additional pathway analysis was done using EggNOG‐mapper (v. 2.1.9) and KEGG Mapper Reconstruct.

### 2.4 Phylogeny Tree Construction

OrthoFinder (v2.5.5; Emms and Kelly 2019), with the msa option selected for alignment and IQtree with 1000 iterations was selected for tree inference, was used to generate a phylogenetic tree of selected MAGs and known acetogens. One representative genome from each genus (23) identified to have known acetogens (Drake et al. 2008; Kim et al. 2023) as well as a few syntrophic acetate‐oxidising bacteria (Singh and Schnürer 2021) was selected for tree construction. This species tree was created with inference from all genes (STAG; Emms and Kelly 2018) and species tree root inference from gene duplication events (STRIDE; Emms and Kelly 2017) with 72 single copy orthologous genes being used. In addition, genomes closely related to the lowest taxonomic classification (by GTDB) of the selected MAGs were also included in the tree. The only representative genome from *Limnochordia*, type‐strain *Limnochordia pilosa*, was included as the closest relative to one of the MAGs. The tree was visualised using FigTree (v. 1.4.4; Rambaut 2018).

## 3 Results

### 3.1 Overall Reactor Community

The investigation of the raw reads with Kraken provided insights into the phylogenetic composition across all three reactors. R1 and R2 shared similar characteristics, with Thermotoga (36% and 11%, respectively) as the dominant bacterial phylum, followed by Bacillota (Firmicutes; 5%; Figure S1A,B). However, in R3, Thermotoga only represented 0.4% of all reads and instead, the community in this reactor was dominated by the phyla Bacillota (9%), *Actinomycetota* (2%) and Proteobacteria (2%; Figure S1C). In addition, some minor phyla were detected. Independent of the reactor, the phylum Thermotoga was represented by *Defluviiotoga tunisiensis* (Figure S1C) while Bacillota was dominated by class *Clostridia* and *Bacilli*, and for R1 and R2, also class *Tisserellia*. R1 and R2 both had 2% of the reads categorised as Archaea in the Euryarcheota phylum. In R1, these reads classified under families *Methanomicrobiaceae* (1%) and *Methanobacteriaceae* (0.9%). In reactor R2, the archaeal community was predominantly composed of *Methanomicrobiaceae*, which accounted for (2%) of the total reads. A smaller fraction of the archaeal population, 0.05%, was identified as *Methanobacteriacae*. R3 had 5% of the reads classified as Archaea in the Euryarcheota phylum, with *Methanosarcinaceae* (3%) as the dominating family, followed by *Methanobacteriaceae* (1%) and *Methanomicrobiaea* (0.2%).

### 3.2 Metagenomic Assembled Genomes Recovery

In all three reactors, between 52% and 67% of all reads were binned, and a total of 109 bins were recovered, with 33, 34, and 42 bins recovered from reactor R1, R2, and R3, respectively (Table 2). The relative abundance based on the number of reads for each bin ranged from 0.034% to 22.7% of total reads (Table S1). Out of the 109 bins, 42 were high‐quality bins considered as MAGs, and 23 were medium‐quality bins. The remaining bins were marked as low quality, characterised by low completeness (< 70%) or exhibited a substantial amount of contamination (> 10%). A detailed analysis can be found in the Tables [Link], [Link] and Figures S1 and S2.

**TABLE 2:** Metagenomic statistics from reactors R1, R2 and R3.

	R1	R2	R3
% of reads binned	67.3	59.1	52.02
High‐quality bins (≥ 90% Completeness, ≤ 10% Contamination)	16	14	14
Medium quality bins (≥ 70% completeness, > 10% contamination)	7	5	9
Low quality bins (< 70% completeness, > 10% contamination)	10	15	19
Total bins	33	34	42
Total reads	30,601,042	24,405,258	24,687,258
Raw reads classified (%)	47.54	24.04	22.39
Raw reads unclassified (%)	52.46	75.96	77.61

### 3.3 Bin Classifications

Out of the 109 bins recovered, eleven bins were too low in quality to be classified, seven bins were classified in the archaeal domain, and 91 bins were classified in the bacterial domain by GTDB.

#### 3.3.1 Bacteria

Out of the 91 bacterial bins, most (72 bins) were classified under Bacillota (Figure S2). For R1 and R2, the classification of the raw reads revealed a high abundance of reads for Thermotoga, 36% and 11% of reads in R1 and R2, respectively (Figure S1A,B) and two bins from each reactor were classified to this phylum. The remaining bins were represented mainly across an additional 4 phyla: Actinobacteriota, Bacteroidota, Caldatribacteriota, and Synergistota. The bins from R1 and R2 shared similar family classifications, with some notable families including *Acetivibrionaceae*, *Acetomicrobiaceae*, *Caldatribacteriaceae*, *Caldicoprobacteraceae*, *Dysgonomonadaceae*, *Petrotogaceae*, *Syntrophomonadaceae*, *Tepidanaerobacteraceae*, *Tepidimicrobiaceae*, and *Thermacetogeniaceae* (Table S2). In addition, two unique bins were recovered from R1 and classified to the family *Tissierellaceae* and *UBA3941* (order *Caldicoprobacterales*), while R2 included unique bins classified to *DTU073* (order *DRI*‐*13*, class *Peptococcia*) and *UBA660* (order *RF39*, class *Bacilli*). Bins recovered from R3 were classified to several of the same families found in the other two reactors but had an additional number of unique bins, classified to the families *Acutalibacteraceae*, *Clostridiaceae*, *Dethiobacteraceae*, *Mycobacteriaceae*, *Peptostreptococcaceae*, and *Turicibacteraceae* (Table S2). Also, bins belonging to several unclassified bacterial families were recovered: *CAG*‐*272*, *DTU030*, *DTU52*, and *DTU083*. In addition, one bin was unique for R3 and R2 but absent from R1, belonging to an unclassified family of *DTU022* (class *Dethiobacteria*).

#### 3.3.2 Methanogens

In reactor R1, three methanogen MAGs were recovered with high completeness (> 97%) and low contamination (> 10%), classified as *Methanothermobacter wolfeii* , *Methanoculleus thermophilus*, and *Methanoculleus thermohydrogenotrophicum* with an ANI similarity of 99.88%, 99.76%, and 99.56%, respectively. In R2, a single methanogen was recovered with a completeness of 98.37% and 0% contamination, *Methanoculleus thermohydrogenotrophicum* (ANI 99.72%). In R3, *Methanothermobacter wolfeii* and *Methanosarcina flavescens* (completeness > 97%, contamination < 1%; ANI 99.67% and 99.8%) were the high‐quality bins that represented the potential methanogenic players. A third bin of low quality was also recovered from this reactor (completeness 22.43%), classified as *Methanobacterium sp000499765*.

### 3.4 Relative Abundance of Bins

In R1 and R2, the most abundant bins (mapped reads) were classified as genus *Defluviitoga*: R1.3 (22.7%), R1.10 (10.8%), R2.18 (20%) and R2.27 (3.5%). In R1, other abundant bins were R1.21 (5.8%), R1.22 (3.2%) and R1.24 (5.8%), belonging to the class *Limnochordia*, class SHA‐98, and order *Bacteroidales*, respectively. In R2, class Limnochordia was represented by R2.8 (5.3%) and an additional abundant bin was R2.33 (4.6%) classified to class *Bacteroidia*. Both R1.24 and R2.33 were identified as *DTU049 sp001512885*. Several bins in lower abundance (1%–4%), such as bin R1.32 and R2.26, were classified as *Tepidanaerobacteraceae*, bin R1.25 was classified as *Syntrophomonadaceae*, and bin R1.8 and R2.32 were classified as *Thermacetogeniaceae*. An unclassified species (JAAZPQ01 sp012797175) from the *Dethiobacteria* class was recovered in reactor R2 (bin R2.15; Table S2). The most abundant methanogenic bin in both R1 and R2 (0.8%–3%) was classified as *Methanoculleus*. In addition, *Methanothermobacter* (0.7%) was also recovered from reactor R1 (R1.28; Table S2).

R3 showed differences in the abundance profile compared to R1 and R2 (Table S1). The most abundant bin was R3.32 (15.4%), which belonged to class Bacteroidia and was classified as *DTU049 sp001512885*. In line with the other investigated reactors, the next two most abundant bins belonged to the class *Limnochordia*, R3.10 (9.4%) and R3.37 (3.1%). In addition, bin R3.12, belonging to the genus *Clostridium*, represented 2.3%, and R3.36 (0.4%), which was further classified to the *Dethiobacteraceae* family (Table S2). The most abundant methanogen was *Methanosarcina flavescens* (R3.42) with 3.1% of the mapped reads, followed by *Methanothermobacter wolfei* (R3.28) representing 0.87% of the reads. The remaining bins that were not specifically mentioned had abundance between 0.03% and 2% (Table S2).

### 3.5 Presence of Pathway Genes

**FIGURE 1:**
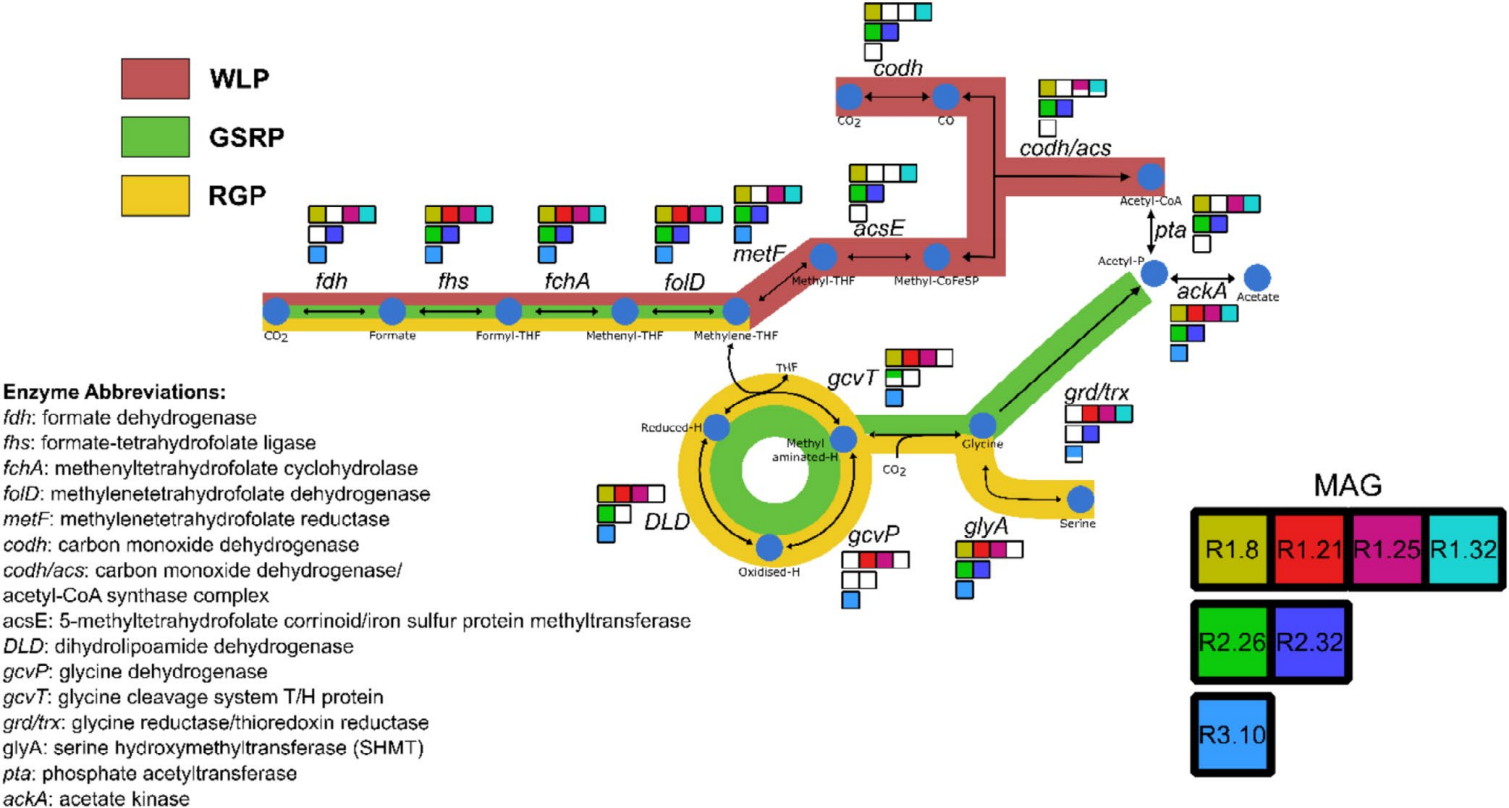
The recovery of the WLP and GSRP genes in the candidate MAGs. Gene presence is indicated by the filled‐in square. A partially filled square indicates partial recovery of an enzyme, i.e. genes encoding for all the subunits of the enzyme were not present.

The initial screening for the WLP key gene, *fhs*, illustrated that nearly all the bins possessed the gene (78 out of 109, Table S3). Further analysis of the high‐quality bins considered as MAGs illustrated that in most cases genes encoding for the first half of the methyl branch of the WLP were also present (Figure 1). However, genes encoding enzymes for the eastern branch, e.g., the *codh* and *codh/acs* complex, could not be observed in most of these high‐quality MAGs. These genes are responsible for the key reactions of reducing CO_2_ to CO and the formation of the carbonyl group of the acetyl‐CoA (Figure 1). Many of the medium and low‐quality bins were also lacking *codh/acs* genes or had only a single component of the complex (Table S3). A further analysis of the unbinned reads revealed a handful of genes that belonged to the *codh/acs* ortholog, but these were not assigned to any bins (Table S3). Collectively, across the three reactors, only five high‐quality MAGs (R1.8, R1.25, R1.32, R2.26 and R2.32) had a complete set or fragments of genes encoding for the *codh/acs* complex (Figure 1). In addition, two additional MAGs, R2.15 and R3.36, that were below the quality cut‐off also possessed a nearly complete WLP, missing only one gene (Table S3). The GSRP and the RGP share the first four genes with the WLP, including the *fhs* gene, and have previously been stated to serve as an alternative or cooperative pathway with the WLP (Song et al. 2020; Figure 1). Thus, each bin was further screened for this pathway, and this expanded analysis illustrated that several MAGs lacking the *codh/acs* possessed either a full (R1.21 and R3.10) or partial GSRP (R1.32, R2.32; Figure 1, Table S3). Full information regarding the WLP/GSRP gene content of the MAGs and the remaining bins can be found in Table S3.

For the recovered methanogen bins R1.5, R1.27, R3.28 and R3.42, all harbour the modules for methanogenesis via both CO_2_ and acetate (Table S5). However, R1.29 had partial modules for both pathways due to the absence of several genes from the gene set coding for tetrahydromethanopterin methyltransferase (Table S3). In addition, in reactor R3, one bin (R3.42) had genes for methylamine methanogenesis (Table S5). R1.27, R3.28, and R3.42 also possessed the acetyl‐CoA module containing *cdhABCDE*. All recovered methanogens from all three reactors possess the module for cofactor F_420_ biosynthesis. Moreover, all methanogens were predicted to have several genes (*TrkA, kefC, kch, msc*) that are involved in potassium‐ion regulation.

### 3.6 Novel Acetogens/SAOBs and Their Phylogenetic Placement

**FIGURE 2:**
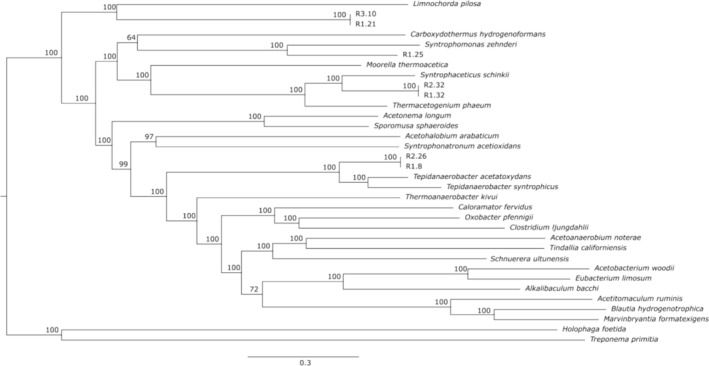
Whole genome sequence tree with acetogen representatives from 23 known genera and several SAOB representatives, constructed with OrthoFinder. The values at the nodes are bootstrap percentages based on 1000 iterations. Bar, 0.3 substitutions per nucleotide position.

Among the high‐quality MAGs, R1.8 and R2.26 displayed a nearly complete WL pathway and thus were selected as potential “true” acetogens. R1.21 and R3.10 were considered as potential acetogens/SAOBs as these MAGs had parts of the WLP and a complete GSRP. Finally, R1.25, R1.32, and R2.32 possessed nearly complete WLP combined with GSRP. According to whole genome comparison using the GTDB database, all these MAGs represented novel species, with classification in most cases only at family level. To position these MAGs in relation to other known acetogens and SAOBs, a phylogeny tree was constructed with species representatives from all known acetogenic genera and SAOB. This illustrated that R1.32 and R2.32 (family *Thermoacetogeniaceae*) were placed close to the known SAOBs *Syntophaceticus schinkii* and *Thermacetogenium phaeum* . Similarly, R1.8 and R2.26 (family Tepidanaerobacteraceae) were clustered close to the syntrophic acid degraders, *Tepidanaerobacter acetatoxydans* and *Tepidanaerobacter syntrophicus* (Figure 2). R1.21 and R3.10 were classified at class rank as Limnochordia and were positioned in the tree closest to *Limnochorda pilosa*.

Two bins, R2.15 and R3.36, were of good quality but did not meet the threshold for high‐quality MAGs. Both bins possessed a near‐complete WLP and were classified as the provisional species *JAAZPQ01 sp012797175* in the Dethiobacteria class and as *DTU027 sp002385745* in the Dethiobacteraceae family, respectively.

### 3.7 Description of New Genus/Species

The genomes presented below are novel species with the ability to produce or consume acetate via WLP/GSRP, which motivated a further analysis to reveal information on their genomic potential and classification as ‘*Candidatus*’ species. The analysis included both an investigation into general genome characteristics and phylogeny as well as analysis involving carbon and energy metabolism (Table S4).

#### 3.7.1 ‘*Candidatus* Thermotepidanaerobacter aceticum’ nov. gen. nov. sp. (R1.8/R2.26)

MAG R1.8 has a length of 2,405,225 bp across 142 contigs with a GC content of 42.24%. This high‐quality MAG has a completeness of 94.06% with 0.96% contamination. MAG R2.26 has a length of 2,068,090 bp in 130 contigs with 42.19% in GC content and 91.35% completeness and 0.96% contamination. MAG R1.8 and MAG R2.26 constituted for 0.75% and 1.9%, respectively, of the total metagenome coverage in the two reactors. R1.8 and R2.26 are considered the same species with 99.84% ANI value. The closest relative in GTDB was represented by another MAG, *DTU063*, with 99.87% ANI. The phylogenetic assessment showed that all three MAGs clustered together and grouped with the known SAOB *Tepidanaerobacter acetatoxydans* (Figure 2). The comparison of genome to genome between R1.8 and *T. acetatoxydans* revealed 72.28% ANI and dDDH between 14.8% and 19.4%; similar ANI and dDDH values were observed in the comparison between R2.26 and DTU063 which were sufficiently low enough to delineate a new genus and species (70% DNA–DNA hybridisation and 95% ANI). MAG R1.8, with the higher completeness, is used and submitted for this ‘*Candidatus*’ proposal. The proposed name is ‘*Candidatus* Thermotepidanaerobacter aceticum’ (Ther.mo.te.pi.da.nae.ro.bac'ter. Gr. masc. adj. thermos, hot; N.L. masc. n. Tepidanaerobacter, a bacterial genus name) aceticum (ace'ti.cum. L. adj. aceticus, of vinegar) and submitted to NCBI under accession JBGJJD000000000.

KEGG pathway analysis illustrated that MAG R1.8 featured complete modules for central carbohydrate metabolism, including glycolysis (Embden‐Meyerhof pathway), glucogenesis, pyruvate oxidation, the non‐oxidative phase of the pentose pathway, and PRPP biosynthesis. Additional carbohydrate metabolism modules included D‐Galacturonate and D‐Glucuronate degradation, and glycogen and nucleotide sugar biosynthesis. Complete sets of genes were recovered for amino acid metabolism and ABC transporters for amino acids, osmoprotectants, and metals (Table S4). MAG R1.8 is the only one of the four ‘*Candidatus*’ species to have phosphotransferase systems (PTS) predicted in the KEGG analysis. In addition to acetate metabolism, as indicated by the presence of most genes in the WLP pathway, it harbours multiple alcohol dehydrogenases, including zinc‐type alcohol and iron‐containing variants, as well as an aldehyde dehydrogenase, potentially involved in propanoate and butanoate metabolism. Energy metabolism pathways include phosphate acetyltransferase (*pta*), acetate kinase (*ackA*), a *rnf* complex, and a complete V/A‐type ATPase module. Cell motility is suggested by the presence of the MS/C ring Type III secretion system.

#### 3.7.2 ‘*Candidatus* Thermosyntrophomonas ammoniaca’ nov. gen. nov. sp. (R1.25)

The length of MAG is 2,396,898 bp over 108 contigs with a 48.39% GC content. R1.25 has a completeness of 97.2% and a contamination of 0.5% and represented ca 1.4% of the total metagenomic reads in R1. R1.25, via GTDB taxonomy, was classified to the family *Syntrophomonoadaceae* and at species level to *DTU018 sp001513155*. R1.25 and *DTU018* were near‐identical with an ANI of 99.82%. On a known taxonomic level, both these MAGs were positioned closest to *Syntrophomonas zehnderi*, however a comparison with R1.25 showed only 67.12% ANI and 10%–15.9% dDDH, which is below the criteria for being the same species. MAG R1.25 was submitted to NCBI under accession number JBGJJE000000000 and proposed as ‘*Candidatus* Thermosyntrophomonas ammoniaca’. Thermosyntrophomonas (Ther.mo.syn.tro.pho.mo‘nas. Gr. masc. adj. thermos, hot; N.L. fem. n. Syntrophomonas, a bacterial genus name) ammoniaca (ammo'nia'ca. L. fem. n. ammonia).

In addition to a near‐complete WLP and phosphate acetyltransferase‐acetate kinase (*pta‐Ack*) pathway for carbon fixation, MAG R1.25 harbours a near‐complete WLP and phosphate acetyltransferase‐acetate kinase (*pta‐Ack*) pathway for carbon fixation, along with complete gene sets for glycolysis, pyruvate oxidation, and the pentose phosphate pathway and metabolism modules for several amino acid metabolism and ABC transporters (Table S4). It also possesses a complete GCS, potentially utilised together with the *pta‐Ack* pathway for acetate metabolism. Consistent with its taxonomic similarity to the lipid‐degrading *Syntrophomonas* (Schink and Muñoz 2014), genes related to beta‐oxidation (acyl‐CoA synthesis) were identified. Moreover, the genome contains genes belonging to alcohol dehydrogenase and aldehyde dehydrogenase, suggesting participation in ethanol and butanol metabolism. While no *ech* hydrogenase or *rnf* complex was found, two of three genes coding for the cytochrome bd complex were predicted. Moreover, an F‐type ATPase and genes for NADH hydrogenase synthesis were predicted. Cell motility is assumed based on the prediction of the MS/C ring Type III secretion system.

#### 3.7.3 ‘*Candidatus* Thermosyntrophaceticus schinkii’ nov. gen. nov. sp. (R1.32/R2.32)

R1.32 had a length of 1,984,119 bp over 163 contigs and 45.54% GC content with completeness of 92.57% and 5.0% contamination. R2.32 had a length of 2,025,861 bp with 140 contigs with a 90.91% completeness and only 0.96% contamination. The GC content was 45.60% The two MAGs are the same species with an ANI of 99.69%. R1.32 and R2.32 constituted 1.2% and 1.4% of the total reads in R1 and R2, respectively. The lowest known taxon‐level classification identified by GTDB was the family Thermacetogeniaceae. The classification at species level was another MAG, *DTU068 sp001513545* with and ANI of 99.8%. *DTU068 sp001513545* had been found in previous metagenomic studies enriched in a high ammonia propionate oxidising culture (Singh et al. 2023) and an enrichment of syntrophic acetate‐oxidising consortia from a thermophilic wastewater treatment plant (McDaniel et al. 2023). The phylogenetic placement of R2.32 clustered with the mesophilic *Syntrophaceticus schinkii*, and the comparison between the two genomes showed 74.03% ANI and 17.5%–22.1% dDDH. R2.32 was predicted with a complete WLP and *pta‐Ack* pathway, potentially utilising F‐type ATPase for ATP synthesis. Genes encoding for *ech* hydrogenases were also found. KEGG modules related to glycolysis, pyruvate oxidation, glycogen and PRPP biosynthesis were observed, along with part of beta‐oxidation module suggesting fatty acid metabolism. The annotation suggests the MAG to have several amino acid metabolisms and membrane transport systems along with some genes encoding for alcohol dehydrogenase and aldehyde ferredoxin oxidoreductase, potentially involved with ethanol metabolism (Table S4). The proposed name is ‘*Candidatus* Thermosyntrophaceticus schinkii’. Thermosyntrophaceticus (Ther.mo.syn.tro.pha.ce'ti.cus. Gr. masc. adj. thermos, hot; N.L. masc. n. Syntrophaceticus, a bacterial genus name) schinkii (schin'ki.i. N.L. gen. n. schinkii, of Schink named after Prof. Bernhard Schinki). The MAG has been uploaded to NCBI and can be found under accession number JBLZGU000000000.

#### 3.7.4 ‘*Candidatus* Thermodarwinisyntropha acetovorans’ nov. gen. nov. sp. (R1.21/R3.10)

MAG R1.21 has a length of 2,037,177 bp over 90 contigs with 59.38% GC content and 91.5% completeness and 2.8% contamination. R3.10 has 2,115,742 bp across 87 contigs with a GC content of 59.32% and a completeness of 94.04% with 2.82% contamination. The relative abundance of R1.21 and R3.10 within their respective reactors was, 5.8% and 9.4% respectively. The ANI between R1.21 and R3.10 was 99.90% and the phylogenetic placement of the two MAGs indicate that they are the same species (Figure 2). MAG R1.21 and R3.10 was classified by GTDB at the species level as an unclassified MAG, *DTU010*, and the next known taxon‐level was class Limnochordia. The only reference representative in this class is *Limnochorda pilosa*. These three MAGs were clustered closest to *Limnocordia pilosa* with an ANI lower than 67%, which is sufficiently low enough to represent a new genus and species. R1.21 and R3.10 was classified to family *DTU010* which recently was proposed as Darwinibacteriaceae (Puchol‐Royo et al. 2023), thus the ‘*Candidatus* Thermodarwinisyntropha acetovorans’, Thermodarwinisyntropha (Ther.mo.dar.wi.ni.syn.tro.pha. Gr. masc. adj. thermos, hot; L. adj. darwinianus, of Darwin; Gr. Adj. syntrophos, living together) acetovorans (ace'to.vo.rans, L.pres. part.acetovorans, consuming vinegar) is proposed and submitted to NCBI under accession number JBGJJF000000000.

‘*Candidatus* T. acetovorans’ annotation revealed GCS genes with *rnf* complex and F‐type ATPase involving carbon and energy metabolism. The KEGG analysis revealed glycolysis, pyruvate oxidation and galactose degradation for carbohydrate metabolism. This genome was also predicted to have several amino acid metabolism and membrane transport system modules (Table S1). This genome also has several genes related to butanoate metabolism and an unclassified alcohol dehydrogenase.

## 4 Discussion

Uncovering the intricacies of the microbial community in thermophilic reactors can facilitate the understanding of the process and ease operation. Thermophilic biogas reactors are susceptible to process disruption due to increased temperature and pH, pushing the equilibrium towards higher ammonia levels and creating inhibitory conditions for microbes (Niu et al. 2015; Gebreeyessus and Jenicek 2016; Westerholm et al. 2018). This genome‐centric metagenomics analysis revealed some of the players in the community of these high ammonia reactors.

### 4.1 Reactor Community

**FIGURE 3:**
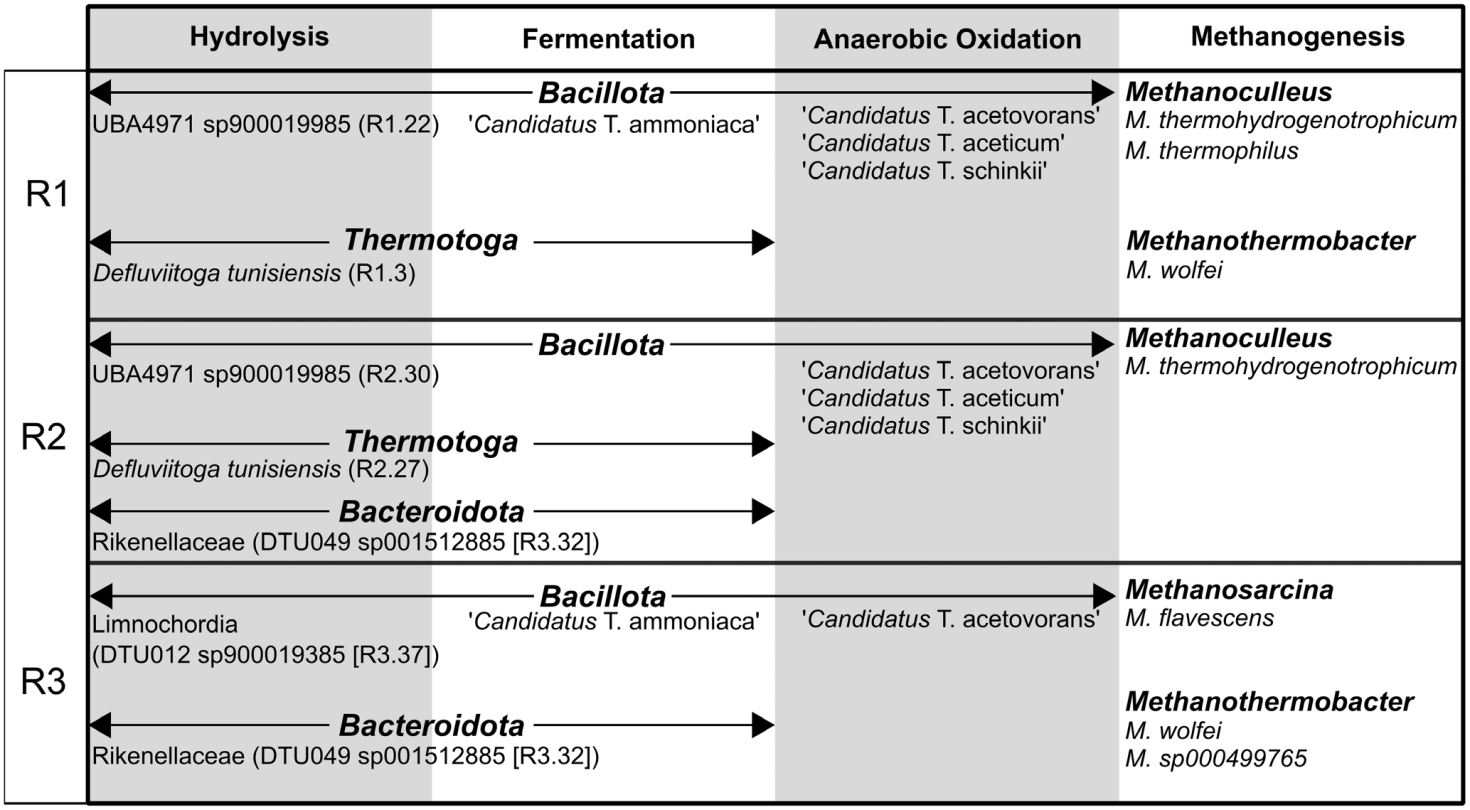
Microbial community overview of abundant players for each phase of anaerobic digestion within each of the reactors.

Several factors can influence the microbial community in a biogas process, with temperature and ammonia levels having a profound impact on both the community compositions and diversity (Yenigün and Demirel 2013; De Vrieze et al. 2015; Pap et al. 2015; Westerholm et al. 2020). The reactors included in the present study were all operated under conditions at high temperature and close to the upper limits regarding ammonia levels (Angelidaki and Ahring 1993; Sung and Liu 2003; Yirong et al. 2017). The observed bacterial communities in the reactors in this study, e.g. dominance by Bacillota (Firmicutes) and Thermotoga phyla with some representation from Bacteroidota (Figure S1), are commonly described in other thermophilic biogas systems, including both moderate and high ammonia processes (Zamanzadeh et al. 2016; Westerholm et al. 2020; Liu et al. 2023). Although the reactors in the present study share many similarities in key parameters influencing the microbial community (Table 1), R3 differed from reactors R1 and R2 in both the bacterial and the archaeal communities (Figures 3 and S1), which may indicate the influence of the chemical composition of the incoming substrate. R1 and R2, both operated on food waste as the main substrate, had considerably more similar microbial compositions compared to R3, which was operated with animal manure and slaughterhouse waste. In line with the results in the present study, dominance of Bacteroidota and Bacillota (Firmicutes) has been found before in other thermophilic biogas reactors utilising swine manure (Tuan et al. 2014; Treu et al. 2018; Murillo‐Roos et al. 2022; Liu et al. 2023) while Bacillota (Firmicutes) and Thermotoga typically dominate in thermophilic food waste reactors (Zamanzadeh et al. 2016; Kim et al. 2018; de Jonge et al. 2020; Zhang et al. 2020). Another influencing factor that could explain the difference between the reactors was the supplement of trace metals. R1 and R2 were supplemented with trace elements and iron to mitigate high H_2_S levels in the outgoing biogas as well as boost microbial activity, while R3 only received iron. It is known that trace elements such as iron, cobalt, and nickel are vital for the optimisation of hydrolysis and acidogenesis, while nickel is critical for acetoclastic and acetogenic reactions as well as the synthesis of cofactor F_420_ (Kim et al. 2003; Choong et al. 2016, 20; Ye et al. 2018). Iron is also an important component for ferredoxin, which is involved in various electron transport reactions (Glass and Orphan 2012). Thermophilic biogas systems are characterised by the dominance of hydrogenotrophic methanogens (Sasaki et al. 2011; Ho et al. 2016; Bu et al. 2018), typically more tolerant to ammonia stress as compared to acetoclastic methanogens (Angelidaki and Ahring 1993). In line with this, the main methanogens in reactors R1 and R2 belonged to *Methanomicrobiales* and *Methanobacteriales* (Figure 3, Tables S1 and S2), also seen to predominate in other food waste digesters (Zamanzadeh et al. 2016; de Jonge et al. 2020). However, in reactor R3, *Methanosarcinales* was instead the more abundant methanogen (Tables S1 and S2). This methanogen is mixotrophic and can grow both with acetate and hydrogen/carbon dioxide (O'Brien et al. 1984; Lackner et al. 2018; Wagner 2020) and thus its function in the present study is unclear. Nevertheless, *Methanosarcina* is commonly found in swine manure digesters (Tuan et al. 2014; Lin et al. 2016; Liu et al. 2023) and has also been reported to tolerate ammonia at levels comparable to those in the present study (De Vrieze et al. 2012).

In all reactors, as illustrated by the total number of reads recovered in the binning process, the most dominating bacterial genera were represented by hydrolytic/fermentative bacteria. The most abundant genus in R1 and R2 was *Defluviitoga* (phylum Thermotoga), represented by the type species *Defluviitoga tunisiensis* . This genus has been proposed to be ammonia tolerant and is often observed as a key hydrolytic bacterium in thermophilic biogas communities (Kim et al. 2018; Westerholm et al. 2018; Treu et al. 2018). The type strain utilises various sugars, cellulose, mannose, and raffinose (Maus et al. 2016) and was likely responsible for the hydrolysis and fermentation steps in R1 and R2 (Figure 3). In R3, the dominating species that was likely fulfilling these functions was represented by *DTU049* (MAG R3.32), being the second most abundant species in R1 and R2 (MAG R1.24 and R2.33). *DTU049 sp001512885* belongs to the *Rikenellaceae* family (phylum Bacteroidota), whose members are known to ferment carbohydrates or proteins, producing acetate, H_2_, and CO_2_ (Abe et al. 2012; Lee et al. 2019). In line with this, the functional analysis of MAG R3.32 identified genes responsible for the metabolism of carbohydrates via glycolysis and acetate production. Moreover, *Rikenellaceae* have been suggested to be a butyrate degrader (Lee et al. 2019). Such metabolism was also indicated in this study by the presence of a complete set of genes for beta‐oxidation in R3.32. *Rikenellaceae* are commonly documented in biogas processes operating on various animal manures and at both mesophilic and thermophilic temperatures (Campanaro et al. 2016; Treu et al. 2018; Koo et al. 2019; Vendruscolo et al. 2020; García Álvaro et al. 2024). Moreover, in line with the dominance in the present study, members within this family have been shown to be ammonia tolerant at high abundance in thermophilic biogas processes at TAN levels of 6.5 g/L (Lee et al. 2019; Puig‐Castellví et al. 2020). Bacillota, one of the dominating phyla across all three reactors based on total reads, was not represented by a single MAG or bin. Instead, it was represented across several low‐abundance bins (< 3%) and three high‐abundance bins: R2.8, R3.10 and R3.37 (5%–9%; Table S1).

In addition to the highly abundant species, several less abundant representatives of the communities were recovered (Figure 3), such as ‘*Candidatus* T. ammoniaca’ (R1.25) in R1 (1.4%). Aside from harbouring the sugar and amino acid metabolism genes (Table S4), ‘*Candidatus* T. ammoniaca’, just like other members of the *Syntrophomonadaceae* family, harbours beta‐oxidation genes responsible for breaking down branched‐chain and short‐chain fatty acids (Table S4; Schink and Muñoz 2014; Narihiro et al. 2016). In R2, R2.15 (3.4%), belonging to *Dethiobacteria* class, was recovered. *Dethiobacteria* was also recovered in R3 (R3.36), but at a much lower abundance (0.4%) and classified further to family level, *Dethiobacteraceae*. In line with the present study, Dethiobacteria has been previously found in thermophilic biogas reactors operating at elevated (3–4 g/L TAN) ammonia levels (Solli et al. 2014; Dyksma et al. 2020; Hashemi et al. 2022; Perman et al. 2022). Members of this class have previously been suggested to include obligate anaerobic, alkaliphilic bacteria that can grow chemolithoautotrophically using the WL pathway for acetate production (Sorokin and Merkel 2022). Accordingly, WLP genes were recovered in both bin R2.15 and R3.36 in the present study (Table S3). However, the type species *Dethiobacter alkaliphilus* utilises acetate with thiosulfate and elemental sulphur as electron acceptors (Sorokin et al. 2008) and thus the recovered bins may possibly be involved in either acetate production or consumption. In R3, *DTU010 sp002391385* (R3.10; 9.4%), was recovered and represented the next most abundant bin in this reactor. This species was also found in R1 and R2 (R1.21 and R2.8), but in lower abundance (< 6%). This provisional species‐level taxon belongs to class *Limnochordia* which was first recovered in a thermophilic reactor operating cattle manure (Campanaro et al. 2016). Since then, *Limnochordia* has been recovered in various biogas digesters using either a co‐digestion of agricultural waste and animal manure or food waste and slaughterhouse waste (Perman et al. 2022; Hassa et al. 2023; Wirth et al. 2023). Within this class the closest known type species is *Limnochorda pilosa*, known to consume various sugars, including glucose, mannose, and fructose, but also proposed as an acetate utilizer (Watanabe et al. 2015). Another species‐level taxon belonging to the class *Limnochordia* was found in all reactors, *DTU012 sp900019385* (R1.16, R2.9, R3.37). Both *DTU010* and *DTU012* have recently been proposed as novel families, *Darwinibacteriaceae* and *Wallaceae*, respectively, belonging to the order *MBA03* in *Clostridia* (Puchol‐Royo et al. 2023). This order has been suggested to be a potential key versatile player in high ammonia and thermophilic biogas systems operating on food waste and manure, not restricted to a single phase of the process and capable of participating in both hydrolysis and acetogenesis (Zheng et al. 2019; Braga Nan et al. 2020; Dyksma et al. 2020; Zeng et al. 2021; Perman et al. 2022; Otto et al. 2023; Puchol‐Royo et al. 2023). Puchol‐Royo et al. (2023) suggested *DTU10* to be potential SAOB, based on the detection of genes of the WLP and GCS. In line with this proposal, *MBA03* have also been documented to co‐occur with hydrogenotrophic methanogens along with other potential SAOB partners, represented by *Syntrophaceticus* and *Dethiobacter* (Otto et al. 2023; Puchol‐Royo et al. 2023). In the present study SAOB candidates (R1.8/R2.26 and R1.32/R2.32), closely related to known mesophilic syntrophic acetate‐oxidising bacterial genera *Tepidanaerobacter* and *Syntrophacticus*, were recovered in R1 and R2, while candidates belonging to *MBA03* were present in all three reactors. All these candidate SAOBs were recovered at a low abundance (< 2%), which seems to be a general trend seen with the population of SAOB (Westerholm et al. 2017). The thermophilic relative to *Syntrophaceticus*, proposed as ‘*Candidatus* Thermosyntrophaceticus schinkii,’ was previously identified in a thermophilic acetate‐enriched chemostat derived from the reactor R1 where it was also suggested to be a SAOB (Westerholm et al. 2018; Singh et al. 2023). The genus *Tepidanaerobacter*, at present, contains two mesophilic representative, one syntrophic lactate degrading bacterium and one syntrophic acetate oxidiser. The thermophilic relative to *Tepidanaerobacter*, ‘*Candidatus* Thermotepidanaeroacter aceticum’, was previously recovered in thermophilic CSTR operating with cattle manure (Campanaro et al. 2016).

In line with the presence of several potential SAOBs, methanogenesis in all three reactors seemed to proceed via SAO, especially in R1 and R2, as indicated by low to no abundance of acetoclastic methanogens and dominance of the hydrogenotrophic methanogens *Methanoculleus* and *Methanothermobacter*. These methanogens are often found to be dominant concurrently with other known and potential SAOB partners: *Tepidanaerobacter*, *MBA03*, *Thermoanaerobacteraceae*, relatives of *Syntrophaceticus schinkii* , and *Spirochaetes* (Westerholm et al. 2010, 2016; Bassani et al. 2015; Müller et al. 2016; Singh et al. 2023). Moreover, these genera are often seen to dominate in high‐ammonia thermophilic biogas systems (Pap et al. 2015; Maus et al. 2016; Bonk et al. 2018). Methanogens have been observed to have varying degrees of ammonia tolerance caused by the inhibitions of gene expression of methanogenesis, cytoplasmic K+ flux, or the thermodynamic limitation placed on the methanogens under ammonia stress (Sprott and Patel 1986; Wang et al. 2015; Yi et al. 2023). Previous studies have reported that ammonia stress can cause an efflux of intracellular potassium (Sprott et al. 1985). Yi et al. (2023) observed the expression of certain genes during ammonia inhibition of *Methanosarcina barkeri* and saw up‐regulated expression of potassium‐ion regulating genes, such as transport system potassium uptake protein (*TrkA*), glutathione‐regulated potassium‐efflux system protein (*kefC*), voltage‐gated potassium channel (*kch*), and conductance mechanosensitive channel (*msc*). The gene prediction analysis of all the recovered methanogens (Table S5) shows the same genes, indicating that these methanogens could potentially regulate their intracellular K^+^ in response to ammonia stress (Yi et al. 2023). Exclusively in R3, the acetoclastic methanogen *Methanosarcina* was recovered at a comparably higher abundance compared to its hydrogenotrophic counterparts, *Methanothermobacter wolfei* and an unclassified species of *Methanobacterium*. Several representatives within the genus *Methanosarcina* can utilise hydrogen in addition to acetate (Thauer et al. 2008; De Vrieze et al. 2012) and are capable of coupling with SAOBs (Ho et al. 2016), as suggested by the genome analysis of MAG R3.42. Increases in the abundance of *Methanosarcina* have also been associated with increases in SAOBs *Tepidanaerobacter*, *MBA03*, and *Thermoanaerobacteraceae* (Zheng et al. 2019). However, in R3, no representatives belonging to either *Tepidanaerobacter* or Thermoanaerobacteraceae were recovered and instead only relatives of *MBA03* (*DTU010* and *DTU012*) representing potential SAOB partners to the *Methanosarcina* representative.

### 4.2 Presence of WLP/GSRP Genes

As per their definition, acetogens utilise the WLP as an energy‐conserving, terminal electron‐accepting, CO_2_‐fixing process. The alternative epithet for the pathway is the reductive acetyl‐CoA pathway, synthesising acetyl‐CoA from C1 compounds (Drake et al. 2008). The WLP consists of two branches, the methyl branch reducing CO_2_ to a C1 carrier (Tetrahydrofolate, THF) via *fdh, fhs*, *folD*, and *metF* (Figure 1); further reduced with another CO_2_ from the carbonyl branch involving *codh* and *acs* to form acetyl‐CoA. The acetyl‐CoA is further synthesised to acetate via a two‐step activation involving the *Pta* and *AckA*. The WLP has also been proposed to operate in the oxidative direction to convert acetate to H_2_ and CO_2_ (Ragsdale and Pierce 2008). Operating the WLP in the oxidation direction, *Pta* and *AckA* are proposed to be used to oxidise acetate, but this two‐step process can be circumvented via acetyl‐CoA synthetase (Hattori 2008; Dyksma et al. 2020; Li et al. 2022). Regarding the SAOBs that have the WLP, some, however not all, possess the entire pathway (Dyksma et al. 2020; Li et al. 2022). Some SAOBs lack the gene for acetyl‐CoA synthetase and are instead proposed to utilise the two‐step activation of acetate or the glycine cleavage system (Li et al. 2022; Puchol‐Royo et al. 2023; Zeng et al. 2024). Li et al. (2022) analysed MAGs recovered from an acetate enrichment originating from a mesophilic high‐ammonia food waste digester and identified two groups of MAGs, one with the conventional WLP, and the other encoding a complete gene pool of the WLP‐GCS pathway. The second group had a higher presence in the reactor, suggesting the importance of the GCS during acetate oxidation (Li et al. 2022). However, the WLP‐GCS pathway has not been exclusive linked to acetate oxidation but has also been observed to be utilised in the reductive direction in acetogens, such as *Clostridium drakei*, co‐utilising both pathways for autotrophic growth (Song et al. 2020). *C. drakei* expressed genes from both WLP and GCS, which elucidated the role of the GCS as a cooperative joint pathway with the WLP, to produce acetyl‐CoA, acetyl‐P, and serine from CO_2_ under varying environmental condition (Song et al. 2020). In the present study none of the recovered bins/MAGs harboured a complete gene set for both WLP and GSRP. In R1 and R2, ‘*Candidatus* T. schinkii’ exhibited a complete WLP and a section of the GSRP, only the *grd*. A close mesophilic relative, *S. schinkii* was previously shown to have all genes needed to encode both the WLP and the GSRP, but GSRP (*grd* or *gcvPAB*) was only expressed when the species were grown at high TAN level (> 3 g/L; Manzoor et al. 2016; Moberg 2020). Possibly, the missing GCS genes in the MAGs recovered in this present study are due to the incompleteness of the genome or perhaps not present in thermophilic relatives of *S. schinkii* (Hattori et al. 2000; Singh et al. 2023). The usage of GSRP and RGP for autotrophic growth has been hypothesised to be dependent on the ammonia concentration in combination with methylene‐H_4_folate to form the H‐protein intermediate in the glycine cleavage system (Kikuchi et al. 2008; Sánchez‐Andrea et al. 2020). The gene *gcvTH*, responsible for the H‐protein transformation step, was predicted in many of the bins and MAGs from all three reactors in the present study, including the potential SAOB ‘*Candidatus* T. acetovorans’ (R3.10/R1.21; Table S3). The ‘*Candidatus* T. aceticum’ exhibited the WLP genes but lacked the formate dehydrogenase, whereas its close relative, *T. acetatoxydans*, harbours a complete set of genes for both the WLP and the GSRP (Westerholm et al. 2011; Müller et al. 2015). ‘*Candidatus* T. ammoniaca’ and ‘*Candidatus* T. acetovorans’ contained GSRP genes and the initial steps of the WLP but lacked *acs*, *codh*, and *metF*, suggesting they may co‐utilise the GCS with parts of the WLP for acetate production via GSRP or RGP (Figure 1). As mentioned previously, the ‘*Candidatus* T. acetovorans’ belongs to *DTU010*, which has been proposed to be a family with SAOBs (Puchol‐Royo et al. 2023). It remains difficult to determine the direction of the pathway, as the presence of the genes do not indicate how they are used. However, known isolated SAOBs that possess the WLP, e.g. *S. schinkii* , *T. phaeum* and *T. acetatoxydans*, have been observed to have NAD(P) transhydrogenase (*PntAB*; Nobu et al. 2015; Manzoor et al. 2016). It has been proposed that SAOBs possess such a gene while homoacetogens lack the *PntAB* (Zeng et al. 2024). These genes were also found in ‘*Candidatus* T. schinkii’, ‘*Candidatus* T. aceticum’ and ‘*Candidatus* T. acetovorans’, potentially supporting them to be SAOB candidates. The ability to utilised either WLP or GSRP might give a possibility to grow at different reduction potentials, as proposed before (Bar‐Even et al. 2012; Song et al. 2020). Both pathways share similar electron carriers, however WLP uses the comparably more reduced Ferrodoxin (*E*'^0^ = −430 mV) while GSRP uses primarily NADPH (*E*'^0^ = −370 mV; Bar‐Even et al. 2012). This would allow CO_2_ fixation via GSRP under limited reduced ferredoxin conditions.

While the definition for acetogens is well‐established, the criteria for SAOB remains relatively flexible. It is difficult to determine whether the retrieved candidates are acetate producers or consumers. The presence of the WLP indicates the potential for acetogenic metabolism, however several indicators—such as the predicted NAD(P) transhydrogenase, the presence of hydrogenotrophic methanogens and the precedent determined by other studies—indicates SAO activity instead. In the case of *MBA03*, it has been hypothesised as a potential SAOB due to the presence of WLP genes that is coupled with the GCS (Puchol‐Royo et al. 2023). The representatives in *MBA03*, including the MAGs recovered in the present study, are missing the *acs*, *CODH*, and *pta*, coding for the carbonyl branch of the WLP, but this branch could be replaced with glycine reductase to allow SAO (Li et al. 2022; Puchol‐Royo et al. 2023). The identity of a SAOB may not solely depend on possessing the whole WLP. Instead, the presence of the WLP/GCS as seen in Li et al. (2022) may support the indication of SAO. While considering the four candidate genomes in the present study, those that lack the carbonyl branch of the WLP, ‘*Ca*. *T. acetovorans*, are likely able to act as SAOB due to the presence of a full GCS and *pntAB*; ‘*Ca*. T. schinkii’ and ‘*Ca*. T. aceticum’ may be considered as SAOB while harbouring the WLP and the *pntAB*.

This study relied solely on the metagenomic approach, which has its limitations. While metagenomics offers valuable insight into community composition and the potential metabolic capabilities, it cannot confirm the actual microbial activity. This limitation could be addressed in future studies through metatranscriptomics or metaproteomic analyses to verify microbial functions. Additionally, metagenomic data could guide microbial isolation and cultivation, providing stronger evidence for their roles within the reactors (Liu et al. 2022).

## 5 Summary

The combined phylogenetic and pathway analyses propose that the three AD processes investigated were driven by a few key players, as illustrated in Figure 3. Interestingly, despite similar environmental and operational conditions, the reactors showed differences in both the bacterial and archaeal communities, proposing influence of the substrate composition. Hydrolysis and fermentation in the two food waste reactors (R1 and R2) were likely driven by *D. tunisiensis*, belonging to the phylum Thermotoga, and some uncharacterised species belonging to Bacillota (MAGs R1.22 and R2.30). In the swine manure process (R3) the dominant hydrolysers and fermenters were likely uncharacterised species belonging to Bacillota (MAG R3.37) and a member Bacteroidota in the *Rikenellaceae* family (R3.32), supposedly fulfilling a similar role as *D. tunisiensis* . In addition, the ‘*Candidatus* T. ammoniaca’ (R1.25), with the potential to degrade long chain fatty acids were present in R1 and R3. Several novel candidatus species had, according to pathway analysis, the potential to perform oxidation of organic acids, such as ‘*Candidatus* T. acetovorans’, ‘*Candidatus* T. aceticum’ and ‘*Candidatus* T. schinkii’, all with the possibility to represent novel SAOB species. In addition, members within order MBA03 were present in all the reactors and Dethiobacteria was found in both the food waste reactors; both groups proposed to also perform SAO. The presence of these potential SAOBs in all reactors highlight the importance of SAO under high ammonia conditions. However, these species can potentially also be engaged in other metabolic processes. As expected from the high ammonia and temperature conditions the dominating methanogens were mostly members hydrogenotrophic genera, *Methanoculleus* and *Methanothermobacter*, but in R3 also the acetoclastic genus *Methanosarcina* were present. Noteworthy was the link between the potential SAOB community and the methanogens, with *Methanoculleus* prevailing in concurrence with ‘*Ca*. T. schinkii’ and ‘*Ca*. T. aceticum’ while the methanogenic partner to ‘*Ca*. T. acetovorans’ was *Methanosarcina* and/or *Methanothermobacter*.

## 6 Conclusions

Among the three investigated thermophilic high‐ammonia reactors, only a few potential acetogens were recovered, all novel, as defined with the presence of the full WL pathway. Among the high‐quality MAGs only ‘*Ca*. T. aceticum’ could be considered a true acetogen. Recent research has highlighted the possibility for acetogens using a combined WL‐GCS pathway, potentially also used in reverse by some SAOBs. In the present study none of the recovered bins/MAGs harboured a complete gene set encoding for both WLP and GSRP, however, some had almost complete pathways and were likely to be acetogens or SAOB, e g ‘*Ca*. T. aceticum’, ‘*Ca*. T. schinkii’ and ‘*Ca*. T. acetovorans’. These species were likely all involved in the acetate metabolism in the investigated reactors, but more experiments are needed to determine whether these pathways are utilised in the reductive or oxidative direction. Considering the breadth of competence of the dominating key players in the reactors and the apparent enrichment of SAOBs, it seems reasonable to assume that fermentative bacteria play the role as acetate producers instead of acetogens.

## Author Contributions

**George B. Cheng:** writing – original draft, writing – review and editing, data curation, investigation, formal analysis, visualization. **Erik Bongcam‐Rudloff:** supervision, writing – review and editing. **Anna Schnürer:** conceptualization, investigation, funding acquisition, writing – original draft, writing – review and editing, supervision, project administration.

## Conflicts of Interest

The authors declare no conflicts of interest.

## Supporting information


**Figure S1.** Sankey breakdown of reads from each reactor. (A) R1 (B) R2 (C) R3. The x‐axis is the taxonomic level denominated by the first letter: D, Domain; P, Phylum; C, Class; O, Order; F, Family; G, Genus; S, Species.
**Figure S2.** Phylum classification of bins from all three reactors. Bacillota was the most recovered phylum.

**Table S1.** Completeness and contamination information for all bins recovered. The relative abundance of each bin was calculated from the number of mapped reads to the bin over total reads.

**Table S2.** Bin classification from GTDB‐tk analysis.

**Table S3.** Gene annotation analysis for the presence of the WLP and GSRP genes in all bins. X and filled in green cell designates the prescenes of the gene/module. – and red cell signifies missing gene/module. The EC numbers used to screen for the genes are found to the right of the table.

**Table S4.** The presence (green cell with an X) and absence (empty red cell) of KEGG modules in the recovered MAGs.

**Table S5.** Enzymes involved with methanation from bins that were classified as methanogens. Analysis was conducted from KEGG mapper reconstruct. X and filled in green cell designates the prescenes of the gene/module. – and red cell signifies missing gene/module.

## Data Availability

The genomic data is uploaded to NCBI under BioProject number PRJNA1117669. The raw reads are stored under accession number SRR29223149 for Reactor 1, SRR29223148 for Reactor 2, and SRR29223147 for Reactor 3. The ‘*Candidatus*’ genomes are stored under BioSample accession: JBGJJF000000000, JBGJJE000000000, JBGJJD000000000, JBLZGU000000000.
