# Correction to ‘Metagenomic Exploration Uncovers Several Novel “*Candidatus*” Species Involved in Acetate Metabolism in High‐Ammonia Thermophilic Biogas Processes’

**DOI:** 10.1111/1751-7915.70217

**Published:** 2025-08-13

Cheng, G. B., E. Bongcam‐Rudloff, and A. Schnürer. 2025. "Metagenomic Exploration Uncovers Several Novel ‘*Candidatus*’ Species Involved in Acetate Metabolism in High‐Ammonia Thermophilic Biogas Processes." *Microbial Biotechnology* 18: e70133. https://doi.org/10.1111/1751‐7915.70133.

In our original publication, we mistakenly acknowledged Prof. Oren for assisting with the naming of the candidate species. While we did discuss general nomenclatural principles with Prof. Oren, the specific names proposed in the original version were developed independently by the authors, and he did not advise on or approve those names. We regret this misrepresentation and in this corrigendum, we present revised naming suggestions that better reflect appropriate taxonomic practices. These revisions have benefited from input by Prof Oren, whose guidance on naming conventions and principles we gratefully acknowledge.

We here present the corrected versions of the protologues for the four new *Candidatus* genera and species proposed in Section 3.7 of the original paper. ‘*Candidatus* Thermotepidanaerobacter aceticum’ have been changed to ‘*Candidatus* Thermotepidanaerobacter aceticus’ and ‘*Candidatus* Thermodarwinisyntropha acetovorans’ have been changed to‘*Candidatus* Thermodarwinisyntropha acetivorans’. Numbers of figures and tables and references cited below refer to that paper. Further information about the taxa can be found in the original publication and its supporting information. In Figure 3, the updated taxonomic names are included in this corrigendum. Furthermore, there was a spelling mistake of the phylum *Thermotogota*. It was previously spelled as *Thermotoga*, which is the genus name. Moreover, the original name of *Metanoculleus thermohydrogenotrophicum* was given in the databases (GTDB/NCBI), while the name later has been updated to‘*Candidatus* Methanoculleus thermohydrogenitrophicus’. Figure 3 has been updated accordingly.

We apologize for this error.

The corrected Section 3.7 should read as:

**3.7 Description of New Genus/Species**

The genomes presented below are novel species with the ability to produce or consume acetate via WLP/GSRP, which motivated a further analysis to reveal information on their genomic potential and classification as ‘Candidatus’ species. The analysis included both an investigation of general genome characteristics and phylogeny as well as analysis involving carbon and energy metabolism (Table S4).

**‘*Candidatus* Thermotepidanaerobacter’ gen. nov**.

Thermotepidanaerobacter (Ther.mo.te.pid.an.ae.ro.bac'ter. Gr. masc. adj. *thermos*, hot; N.L. masc. n. *Tepidanaerobacter*, a bacterial genus name; N.L. masc. n. ‘*Candidatus* Thermotepidanaerobacter’, a hot‐loving *Tepidanaerobacter*).

The properties of the genus are as the properties of the only species described thus far, ‘*Candidatus* Thermotepidanaerobacter aceticus’.

Type species: ‘*Candidatus* Thermotepidanaerobacter aceticus’

**‘Candidatus Thermotepidanaerobacter aceticus’ sp. nov**.

Aceticus (a.ce'ti.cus. L. neut. n. *acetum*, vinegar; L. masc. adj. suff. ‐*icus*, suffix used with the sense of pertaining to; N.L. masc. adj. *aceticus*, pertaining to vinegar, intended to mean producing acetate).

This taxon is represented by MAG R1.8/R2.26. MAG R1.8 has a length of 2,405,225 bp across 142 contigs with a GC content of 42.24%. This high‐quality MAG has a completeness of 94.06% with 0.96% contamination. MAG R2.26 has a length of 2,068,090 bp in 130 contigs with 42.19% in GC content and 91.35% completeness and 0.96% contamination. MAG R1.8 and MAG R2.26 constituted 0.75% and 1.9%, respectively, of the total metagenome coverage in the two reactors. R1.8 and R2.26 are considered the same species with a 99.84% ANI value. The closest relative in GTDB was represented by another MAG, DTU063, with a 99.87% ANI. The phylogenetic assessment showed that all three MAGs clustered together and grouped with the known SAOB Tepidanaerobacter acetatoxydans (Figure 2). The comparison of genome to genome between R1.8 and T. acetatoxydans revealed a 72.28% ANI and dDDH between 14.8%–19.4%; similar ANI and dDDH values were observed in the comparison between R2.26 and DTU063, which were sufficiently low enough to delineate a new genus and species (70% DNA‐DNA hybridisation and 95% average nucleotide identity). KEGG pathway analysis illustrated that MAG R1.8 featured complete modules for central carbohydrate metabolism including glycolysis (Embden‐Meyerhof pathway), glucogenesis, pyruvate oxidation, the non‐oxidative phase of the pentose pathway, and PRPP biosynthesis. Additional carbohydrate metabolism modules included D‐Galacturonate and D‐Glucuronate degradation, and glycogen and nucleotide sugar biosynthesis. Complete sets of genes were recovered for amino acid metabolism and ABC transporters for amino acids, osmoprotectants, and metals (Table S4). MAG R1.8 is the only one of the four ‘*Candidatus*’ species to have phosphotransferase systems (PTS) predicted in the KEGG analysis. In addition to acetate metabolism, as indicated by the presence of most genes in the WLP pathway, it harbours multiple alcohol dehydrogenases, including zinc‐type alcohol and iron‐containing variants, as well as an aldehyde dehydrogenase, potentially involved in propanoate and butanoate metabolism. Energy metabolism pathways include phosphate acetyltransferase (*pta*), acetate kinase (*ackA*), a *rnf* complex, and a complete V/A‐type ATPase module. Cell motility is suggested by the presence of the MS/C ring Type III secretion system.

Nomenclatural type: ‘*Candidatus* Thermotepidanaerobacter aceticus’ MAG R1.8, submitted to NCBI under accession JBGJJD000000000.

**‘*Candidatus* Thermosyntrophomonas’ gen. nov**.

*Thermosyntrophomonas* (Ther.mo.syn.tro.pho.mo'nas. Gr. masc. adj. *thermos*, hot; N.L. fem. n. *Syntrophomonas*, a bacterial genus name; N.L. fem. n. ‘*Candidatus* Thermosyntrophomonas’, a hot‐loving *Syntrophomonas*).

The properties of the genus are as the properties of the only species described thus far, ‘*Candidatus* Thermosyntrophomonas ammoniaca’.

Type species: ‘*Candidatus* Thermosyntrophomonas ammoniaca’.

**‘Candidatus Thermosyntrophomonas ammoniaca’ sp. nov**.

*ammoniaca* (am.mo.ni.a'ca. N.L. fem. n. *ammonia*, ammonia; N.L. fem. adj. *ammoniaca*, pertaining to ammonia).

This taxon is represented by MAG R1.25. The length of MAG is 2,396,898 bp over 108 contigs with a 48.39% GC content. R1.25 has a completeness of 97.2% and a contamination of 0.5% and represented ca 1.4% of the total metagenomic reads in R1. R1.25, via GTDB taxonomy, was classified to the family *Syntrophomonadaceae* and at species level to DTU018 sp001513155. R1.25 and DTU018 were near‐identical with an ANI of 99.82%. On a known taxonomic level, both these MAGs were positioned closest to *Syntrophomonas zehnderi*; however, a comparison with R1.25 showed only 67.12% ANI and 10%–15.9% dDDH, which is below the criteria for being the same species. In addition to a near‐complete WLP and phosphate acetyltransferase‐acetate kinase (*pta‐Ack*) pathway for carbon fixation, MAG R1.25 harbours a near‐complete WLP and phosphate acetyltransferase‐acetate kinase (*pta‐Ack*) pathway for carbon fixation, along with complete gene sets for glycolysis, pyruvate oxidation, and the pentose phosphate pathway and metabolism modules for several amino acid metabolism and ABC transporters (Table S4). It also possesses a complete GCS, potentially utilised together with the *pta‐Ack* pathway for acetate metabolism. Consistent with its taxonomic similarity to the lipid‐degrading *Syntrophomonas* (Schink and Muñoz 2014), genes related to beta‐oxidation (acyl‐CoA synthesis) were identified. Moreover, the genome contains genes belonging to alcohol dehydrogenase and aldehyde dehydrogenase, suggesting participation in ethanol and butanol metabolism. While no *ech* hydrogenase or *rnf* complex was found, two of three genes coding for the cytochrome bd complex were predicted. Moreover, a F‐type ATPase and genes for NADH hydrogenase synthesis were predicted. Cell motility is assumed based on the prediction of the MS/C ring Type III secretion system.

Nomenclatural type: ‘*Candidatus* Thermosyntrophomonas ammoniaca’ MAG R1.25, submitted to NCBI under accession number JBGJJE000000000.

**‘*Candidatus* Thermosyntrophaceticus’ gen. nov**.

Thermosyntrophaceticus (Ther.mo.syn.troph.a.ce'ti.cus). Gr. masc. adj. *thermos*, hot; N.L. masc. n. *Syntrophaceticus*, a bacterial genus name; N.L. masc. n. ‘*Candidatus* Thermosyntrophaceticus’, a hot‐loving *Syntrophaceticus*.

The properties of the genus are as the properties of the only species described thus far, ‘*Candidatus* Thermosyntrophaceticus schinkii’.

Type species: ‘*Candidatus* Thermosyntrophaceticus schinkii’.

**‘*Candidatus* Thermosyntrophaceticus schinkii’ sp. nov**.

Schinkii (schin'ki.i. N.L. gen. n. *schinkii*, of Schink, named after Prof. Bernhard Schink).

This taxon is represented by MAGs (R1.32/R2.32). R1.32 had a length of 1,984,119 bp over 163 contigs and 45.54% GC content with completeness of 92.57% and 5.0% contamination. R2.32 had a length of 2,025,861 bp with 140 contigs, with a 90.91% completeness and only 0.96% contamination. The GC content was 45.60%. The two MAGs are the same species with an ANI of 99.69%. R1.32 and R2.32 constituted 1.2% and 1.4% of the total reads in R1 and R2, respectively. The lowest known taxon‐level classification identified by GTDB was the family *Thermacetogeniaceae*. The classification at the species level was another MAG, DTU068 sp001513545, with an ANI of 99.8%. DTU068 sp001513545 had been found in previous metagenomic studies enriched in a high ammonia propionate oxidising culture (Singh et al. 2023) and an enrichment of syntrophic acetate‐oxidising consortia from a thermophilic wastewater treatment plant (McDaniel et al. 2023). The phylogenetic placement of R1.32 clustered with the mesophilic Syntrophaceticus schinkii, and the comparison between the two genomes showed 74.03% ANI and 17.5%–22.1% dDDH. R1.32 was predicted to have a complete WLP and pta‐Ack pathway, potentially utilising F‐type ATPase for ATP synthesis. Genes encoding ech hydrogenases were also found. KEGG modules related to glycolysis, pyruvate oxidation, glycogen and PRPP biosynthesis were observed, along with part of the beta‐oxidation module suggesting fatty acid metabolism. The annotation suggests the MAG to have several amino acid metabolisms and membrane transport systems along with some genes encoding alcohol dehydrogenase and aldehyde ferredoxin oxidoreductase, potentially involved in ethanol metabolism (Table S4).

Nomenclatural type: ‘*Candidatus* Thermosyntrophaceticus schinkii’ MAG R1.32, submitted to NCBI under accession number JBGJJD000000000.

**‘*Candidatus* Thermodarwinisyntropha’ gen. nov**.

Thermodarwinisyntropha (Ther.mo.dar.wi.ni.syn.tro'pha. Gr. masc. adj. *thermos*, hot; Gr. masc. adj. *syntrophos*, living together; N.L. fem. n. ‘*Candidatus* Thermodarwinisyntropha’, a hot‐loving syntrophic organism named after Charles Darwin).

The properties of the genus are as the properties of the only species described thus far, ‘*Candidatus* Thermodarwinisyntropha acetivorans’.

Type species: ‘*Candidatus* Thermodarwinisyntropha acetivorans’.

**‘*Candidatus* Thermodarwinisyntropha acetivorans’ sp. nov**.

Acetivorans (a.ce.ti.vo'rans. L. neut. n. *acetum*, vinegar; L. pres. part. *vorans*, devouring; N.L. fem. part. adj. *acetivorans*, acetate‐consuming).

This taxon is represented by MAGs R1.21/R3.10. MAG R1.21 has a length of 2,037,177 bp over 90 contigs with 59.38% GC content and 91.5% completeness and 2.8% contamination. R3.10 has 2,115,742 bp across 87 contigs with a GC content of 59.32% and a completeness of 94.04% with 2.82% contamination. The relative abundance of R1.21 and R3.10 within their respective reactors was 5.8% and 9.4% respectively. The ANI between R1.21 and R3.10 was 99.90% and the phylogenetic placement of the two MAGs indicates that they are the same species (Figure 2). MAG R1.21 and R3.10 were classified by GTDB at the species level as an unclassified MAG, DTU010, and the next known taxon‐level was class *Limnochordia*. The only reference representative in this class is *Limnochorda pilosa*. These three MAGs were clustered closest to *Limnocordia pilosa* with an ANI lower than 67%, which is sufficiently low enough to represent a new genus and species. R1.21 and R3.10 were classified to family DTU010 which recently was proposed as *Darwinibacteriaceae* (Puchol‐Royo et al. 2023). ‘*Candidatus* T. acetivorans’ annotation revealed GCS genes with rnf complex and F‐type ATPase involving carbon and energy metabolism. The KEGG analysis revealed glycolysis, pyruvate oxidation and galactose degradation for carbohydrate metabolism. This genome was also predicted to have several amino acid metabolism and membrane transport.

Figure 3, with the updated taxonomic names included, should read as:

**FIGURE 3:**
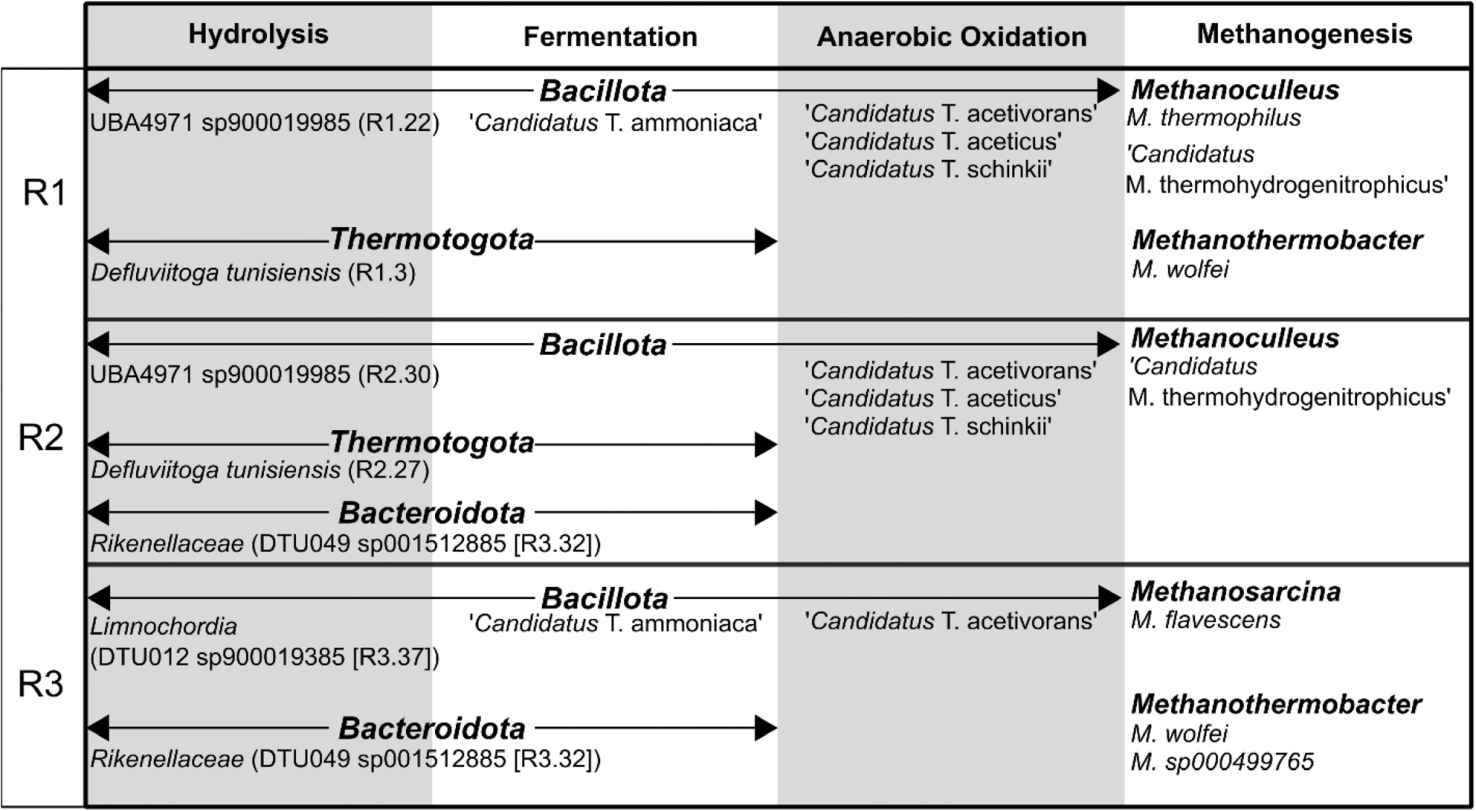
Microbial community overview of abundant players for each phase of anaerobic digestion within each of the reactors.

