# Supplementary material for: Metagenomic Exploration Uncovers Several Novel ‘Candidatus’ Species Involved in Acetate Metabolism in High‐Ammonia Thermophilic Biogas Processes
Source: Microb Biotechnol. 2025 Mar 24;18(3):e70133. doi: 10.1111/1751-7915.70133 (PMC11932165; doi:10.1111/1751-7915.70133)
Supplement: Supplementary file 1 — Figure S1. Sankey breakdown of reads from each reactor. (A) R1 (B) R2 (C) R3. The x‐axis is the taxonomic level denominated by the first letter: D, Domain; P, Phylum; C, Class; O, Order; F, Family; G, Genus; S, Species. Figure S2. Phylum classification of bins from all three reactors. Bacillota was the most recovered phylum. [file MBT2-18-e70133-s002.docx]

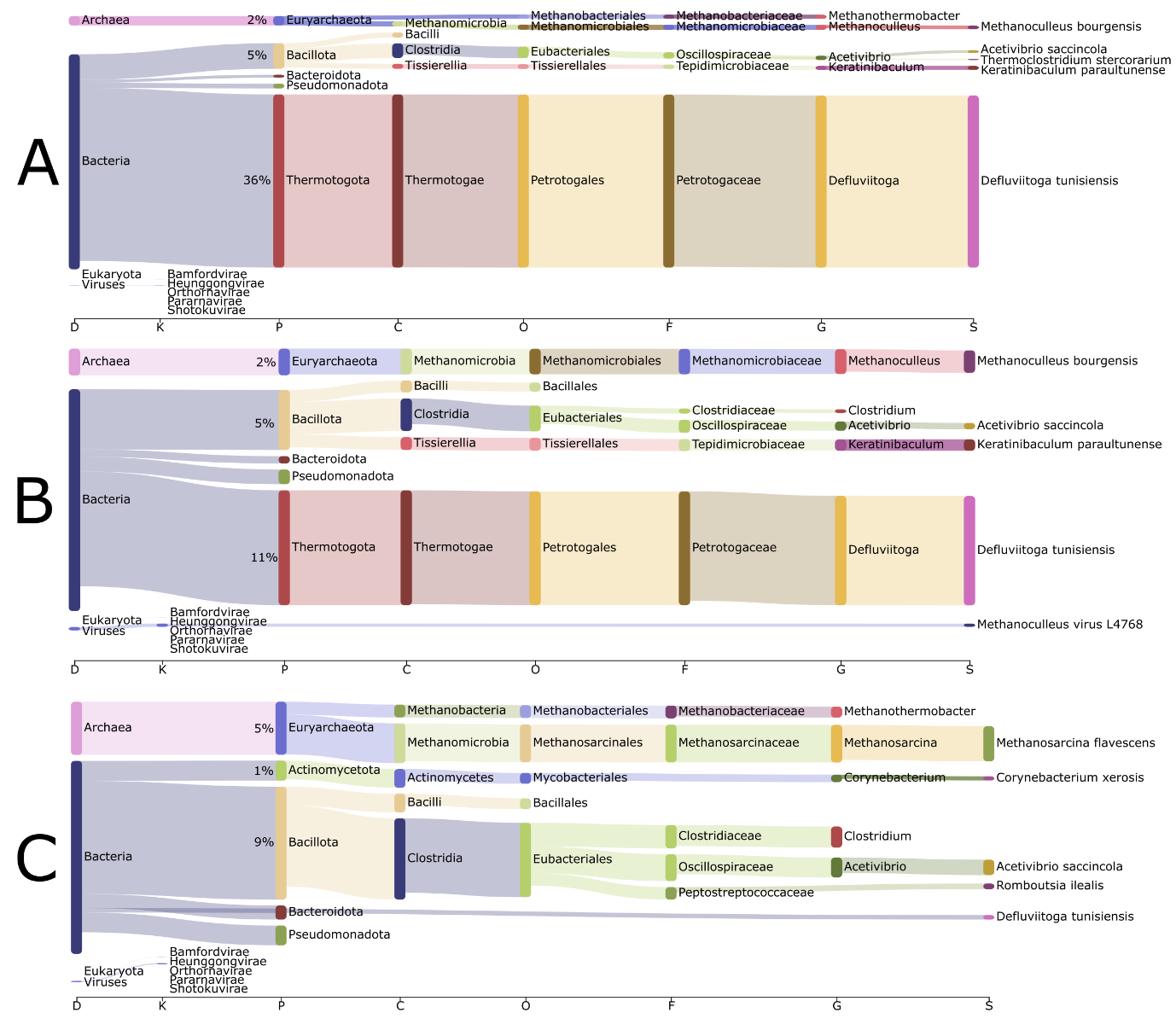


Supplementary Figure 1 Sankey breakdown of reads from each reactor. A) R1 B) R2 C) R3. The x-axis is the taxonomic level denominated by the first letter, D, Domain; P, Phylum; C, Class; O, Order; F, Family; G, Genus; S, Species.


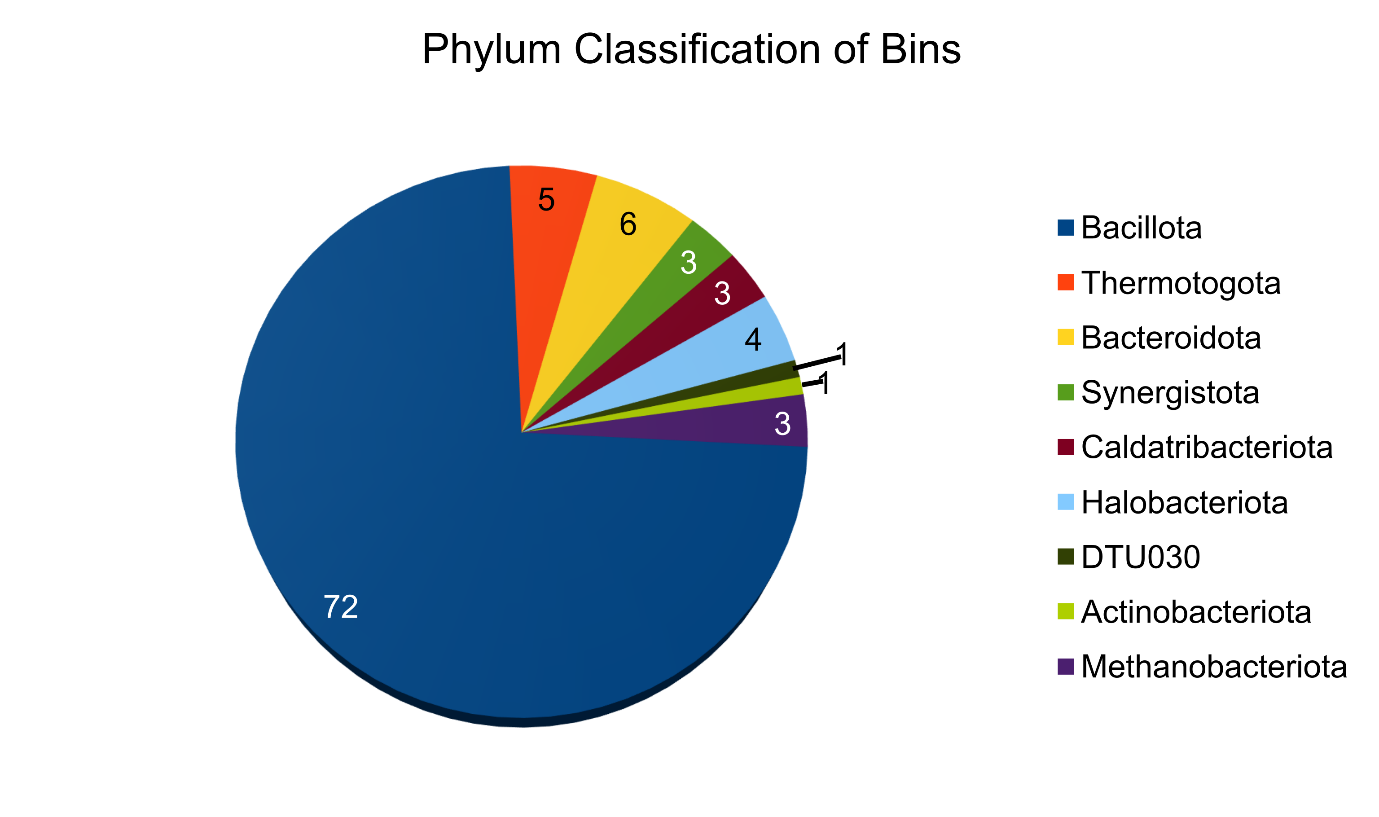


Supplementary Figure 2 Phylum classification of bins from all three reactors. Bacillota was the most recovered phylum.
